# Anxiety-potentiated amygdala–medial frontal coupling and attentional control

**DOI:** 10.1038/tp.2016.105

**Published:** 2016-06-07

**Authors:** O J Robinson, M Krimsky, L Lieberman, K Vytal, M Ernst, C Grillon

**Affiliations:** 1 University College London, Institute of Cognitive Neuroscience, London, UK; 2 Section on Neurobiology of Fear and Anxiety, National Institute of Mental Health, National Institutes of Health, Bethesda, MD, USA

## Abstract

Anxiety disorders can be treated both pharmacologically and psychologically, but many individuals either fail to respond to treatment or relapse. Improving outcomes is difficult, in part because we have incomplete understanding of the neurobiological mechanisms underlying current treatments. In a sequence of studies, we have identified ‘affective bias-related' amygdala–medial cortical coupling as a candidate substrate underlying adaptive anxiety (that is, anxiety elicited by threat of shock in healthy individuals) and shown that it is also chronically engaged in maladaptive anxiety disorders. We have provided evidence that this circuit can be modulated pharmacologically, but whether this mechanism can be shifted by simple psychological instruction is unknown. In this functional magnetic resonance imaging study, we extend a previously used translational anxiety induction (threat of shock) in healthy subjects (*N*=43) and cognitive task to include an element of instructed attentional control. Replicating our previous findings, we show that induced anxiety engages ‘affective bias-related' amygdala-dorsal medial frontal coupling during the processing of emotional faces. By contrast, instructing subjects to attend to neutral shapes (and ignore faces) disengages this circuitry and increases putative ‘attentional control-related' coupling between the amygdala and a more rostral prefrontal region. These neural coupling changes are accompanied by corresponding modulation of behavioural performance. Taken together, these findings serve to further highlight the potential role of amygdala–medial frontal coupling in the pathogenesis of anxiety and highlight a mechanism by which it can be modulated via psychological instructions. This, in turn, generates hypotheses for future work exploring the mechanisms underlying psychological therapeutic interventions for anxiety.

## Introduction

Anxiety disorders are debilitating conditions that carry enormous individual, social and economic costs.^1, 2^ Current treatments are unfortunately not as effective as we would wish. Many anxious individuals are treatment non-responders and those who do respond frequently relapse.^3, 4^ Understanding the neurocircuitry of adaptive and maladaptive anxiety may eventually aid in the development of better, more biologically informed, preventative and treatment approaches. In this study, we therefore take a neural mechanism that we have previously linked to both normal and pathological anxiety,^5, 6, 7, 8^ and which we have shown to be modulated pharmacologically,^7^ and explore whether it can be modulated psychologically through attentional control.^9, 10^

Our programmatic line of studies suggests that pathological anxiety arises, at least in part, from a failure to downregulate ‘adaptive' anxiety mechanisms that promote negative affective bias,^11^ such that anxious individuals are inappropriately primed for threats where none are apparent.^5, 6, 7, 8^ Such adaptive anxiety can be reliably induced in humans using the threat of unpredictable shock paradigm.^11, 12^ Using this technique (which is translated from animal models^13^), we have built on prior work implicating the dorsal anterior (mid)cingulate/medial frontal cortex^14, 15^ and amygdala^16^ in anxiety, to show that induced anxiety increases coupling between these regions as a circuit^5, 8^ (alongside behavioural indices of negative affective bias on face identification tasks^5, 11^). At the same time, this mechanism is engaged chronically and inappropriately (that is, in the absence of experimental threat) in patients with anxiety disorders.^6^ Moreover, increased engagement of this circuit is seen along a spectrum in those displaying the highest trait anxiety,^5, 6, 8^ thus providing a link between underlying neural processes and observed clinical symptomatology. Taken together, these findings suggest that changes to this circuit may constitute a neural mechanism from which a diagnosis of pathological anxiety emerges.

Our main clinical goal, however, is to normalise such aberrant mechanisms through treatment. The primary treatments for anxiety disorders are serotonergic medication and psychological therapy.^17^ We have shown that the neurotransmitter serotonin can modulate this circuit.^7^ Specifically, reducing serotonin function in healthy individuals serves to release activity within this circuit in the absence of threat: mimicking pathological anxiety.^7^ Restoring inhibition of this circuitry in anxious individuals may therefore be one mode of action of serotonergic medication in patients.^7^

Psychological treatments of anxiety disorders can, however, be as effective as pharmacological treatment^18^ and are in fact recommended as a first-line intervention in the UK.^17^ An unanswered question, therefore, is whether it is possible to modulate activity within this established amygdala–medial frontal circuitry psychologically, without pharmacological intervention. One goal of a number of psychological treatments is to improve top–down attentional control,^9, 10^ a process which is thought to be impaired in anxiety disorders.^19, 20^ The impact of attentional control over the threat-mediated neural circuitry we have identified is, however, unknown. A number of studies have shown altered activity (that is, not connectivity) in the dorsal medial prefrontal/cingulate cortex and amygdala during attentional control,^9, 18, 21, 22, 23^ and indeed dorsal prefrontal activation has been implicated in attentional-control-related treatment outcomes in anxiety disorders.^9, 24^ However, whether this extends to threat-induced circuit coupling is at present unknown.

Interestingly, many of the same regions implicated in meta-analyses of attentional control^9^ overlap with those seen in meta-analyses of threat or anxiety.^14, 15^ As such, it has been hypothesised that anxiety and attention are linked because their underlying neural processes compete for limited neural resources and that attentional control mechanisms dampen anxiety processes by beating them in a competition for neural processing space.^25^ Direct evidence for this proposition and whether it pertains to circuit coupling is, however, lacking.

In this study, we therefore added an ‘attentional control' component to our previously used face identification task.^5^ We created compound stimuli comprising non-emotional distractors (shapes) behind fearful or happy faces. On half the task, subjects were asked to perform the task as before^5^—attend to the emotion of the face (albeit this time ignoring the shapes which were not present in the prior task)—while the remaining task required subjects to ignore the faces and attend to the shapes. In this latter condition, the faces acted as task-irrelevant emotional distractors.^23, 26^ Subjects were thus required to engage attentional control mechanisms to process the shapes while preventing interference from the faces (although prior work suggests unattended faces may still ‘leak through' to partially impact performance^26, 27^). Both task phases were again completed while subjects underwent threat of unpredictable shock and when they were safe from shock. In other words, we asked whether, in healthy controls, attentional control can modulate threat of shock-potentiated coupling within medial frontal-amygdala circuitry.^5, 6, 7, 8^

The specific prediction emerging from our prior work is that attending to emotional faces will engage anxiety-potentiated ‘affective bias-related' medial frontal-amygdala circuitry,^5, 6, 7, 8^ while disengaging from the faces and attending to neutral shape stimuli will correspondingly disengage this circuitry as healthy individuals practise top down attentional control.^19^ Indeed attentional control and affective bias may recruit overlapping medial frontal-amygdala circuitry.^25^ Support for this hypothesis would provide the first evidence that this threat-potentiated circuit, linked to pathological anxiety,^6^ and subject to pharmacological control,^7^ can also be subject to psychological attentional control.

## Materials and methods

### Recruitment

Healthy control participants (*N*=50) were recruited in response to advertisements (newspaper and public transport) in the Washington, DC metropolitan area. Following an initial telephone screen, participants visited the National Institute of Health (NIH) for comprehensive screening by a clinician, which comprised a physical examination, urine screen and a Structured Clinical Interview (SCID) from the Diagnostic and Statistical Manual of Mental Disorders (DSM), Fourth Edition.^28^ Exclusion criteria were contraindicated medical disorder (that is, those thought to interfere with brain function and/or behaviour), past or current psychiatric disorders and use of psychoactive medications or recreational drugs (per urine screen). *N*=43 (18 male; 27 white, 11 black, 5 Asian; mean age 25±5 (s.d.; age range 20–44)) of the original 50 were included in final analysis (*N*=7 excluded; 3 due to scan artefacts; 4 due to behavioural task acquisition issues). All participants provided written informed consent and received compensation for taking part in the study. The Combined Neuroscience Institutional Review Board of the NIH approved the study protocol.

### Manipulation

A Digitimer DS7A constant current stimulator (Digitimer, Welwyn Garden City, UK) was used to administer shocks to the top of the left foot using 2 Ag/AgCl electrodes (6 mm). Shock intensity was determined individually during a workup procedure performed in the scanner before scanning. Subjects retrospectively rated their anxiety during the threat and safe blocks on a 10-point scale. Data were analysed in a scan × shock-threat analysis of variance (ANOVA) for individuals (*N*=36) with complete ratings across both scans.

### Task

**Figure 1:**
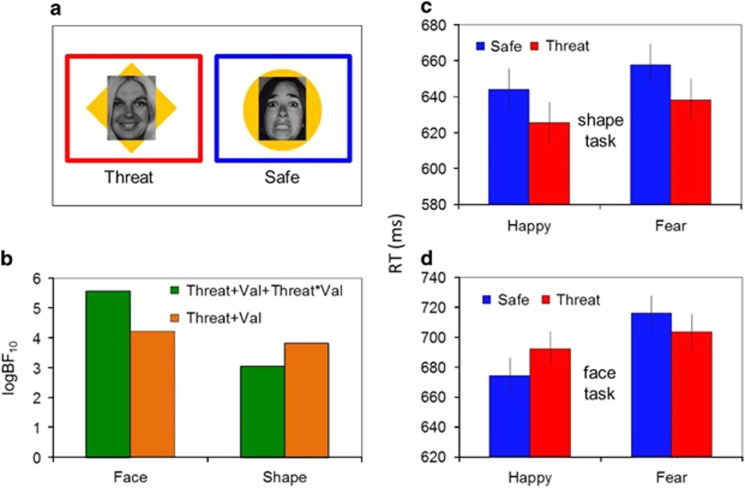
(**a**) Example stimuli; happy/diamond in shock-threat condition on left and fear/circle in safe condition on right (all stimuli counterbalanced across conditions). (**b**) A visual representation of Bayesian model evidence for the behavioural analysis, showing the replicated shock-threat × valence interaction in face task (that is, the green bar is higher) and main effect of shock-threat and main effect of valence in shape task (that is, the orange bar is higher). (**c**) Reaction times in shape task and (**d**) face task (bars represent s.e.m.).

The task was modified from that used previously^5^ to encompass shapes behind the faces (Figure 1a). Each attention block (order counterbalanced) was introduced with either ‘This is the FACE version of the task' in which subjects responded to the emotion (fear/happy) of the face or ‘This is the SHAPE version of the task' in which subjects responded to the shape (circle/diamond). Reaction time and accuracy were measured for these shape or face responses. A jitter of 4–6 s was included between blocks. During both blocks, the image was presented for 1 s, followed by a jittered 4–2-s inter trial interval consisting of a fixation cross ‘+'. Within each block, the task alternated between safe and threat conditions (4 per attention block) consisting of 10 trials per condition (160 total; 80 per attention block). A blue border surrounded the safe condition, and a red border surrounded the threat condition (Figure 1a). This entire sequence was completed twice in two separate scan acquisitions with a short break in between in which retrospective ratings were completed verbally over the scanner intercom. Two shocks were administered pseudo randomly in two of the threat conditions. Thirty seconds of fixation was included at the start and end of each acquisition to act as implicit baseline. Stimuli were displayed on a projector screen viewed by means of a mirror in the head coil.

### Behavioural analyses

Outcomes of interest were analysed using complementary frequentist (SPSS, IBM, Armonk, NY, USA; released 2013. IBM SPSS Statistics for Windows, Version 22.0, IBM) and Bayesian (JASP^29, 30, 31^) omnibus ANOVAS comprising task × shock-threat × valence × shape interactions before being broken down into constituent effects. Bayesian analyses were included as they have a number of advantages including enabling a more fine-grained model comparison approach (for example, it is possible to determine how much better or worse a given model of the data is relative to another and also makes it possible to accept the null hypothesis over a given model). We refer readers as unfamiliar with this approach to the following tutorial;^32^ but briefly put, a Bayes factor (BF) quantifies the evidence for one hypothesis relative to another. As many researchers may be unfamiliar with this approach, however, we also include the same tests using frequentist statistics for comparison. For frequentist tests, *P<*0.05 was deemed significant; for Bayesian analyses, BFs were calculated utilising a default Cauchy prior^30^ to determine how much better (or worse) models representing interactions of interest were relative to the null model (of subject). Models containing interactions include the main effects of all the individual factors within the interactions. The ‘winning' model was defined as the single model with the highest BF relative to the null (BF10 (note that the ‘BF10' nomenclature refers to the BF for H1 vs H0 [model relative to null], as distinct from ‘BF01,' which is the BF for H0 vs. H1 [null relative to model]. It does not refer to log10. Where we refer to ‘logBF10,' we report the natural log of the BF10 values. This log is not required; it is simply used to make the frequently very large numbers more interpretable.)). The natural log of these numbers was then taken to improve interpretability (logBF10). As a rule of thumb,^33^ a logBF10 of 1–2.3 is substantial evidence, >4.6 is decisive, whereas <1 is evidence in favour of the null.

### Functional Magnetic Resonance Imaging

We used a 3T Skyra scanner (Siemens, Malvern, PA, USA) to acquire two 433 volume acquisition echo planar imaging sequence (flip angle 70° repetition time (TR) 2000 ms; echo time (TE) 30 ms; field-of-view (FOV) 100 cm; slice thickness 3 mm; matrix 64 × 64 samples sagittal). We discarded the first five volumes from each run to allow for scanner equilibration. The structural sequence comprised a magnetisation-prepared rapid gradient echo anatomical reference image (flip angle 9° TR 1900 ms; TE 2.1; inversion time 450 ms; FOV 100 cm; slice thickness 0–9 mm; matrix 256 × 256). We pre-processed and analysed images using SPM version 8 (Wellcome Trust Centre for Neuroimaging, London, UK). Structural images were used for localisation and coregistration of the functional data (pre-processing steps were replicated from prior work^5, 6^ and consisted of realignment, coregistration, segmentation, normalisation and smoothing. For full specification see code: https://dx.doi.org/10.6084/m9.figshare.3144862.v1). Six motion parameters were included as regressors of no interest; shock events were modelled and excluded from analysis. Event related analysis was completed by taking a contrast representing all trial types into a flexible factorial design collapsed across the shapes into an attention × shock-threat × valence design matrix. Connectivity analysis was completed taking all trial types into a generalised psychophysiological interaction (gPPI) analysis using the gPPI toolbox^34^ using the anatomically defined right amygdala as a seed region (as before^5, 6, 7^). The connectivity estimates for all trial types were then analysed using flexible factorial design collapsed across the shapes into an equivalent design matrix as the event-related analysis. Connectivity and event-related analyses were both computed using *t*-tests and broken down into component parts.

### Regions-of-interest

**Figure 2:**
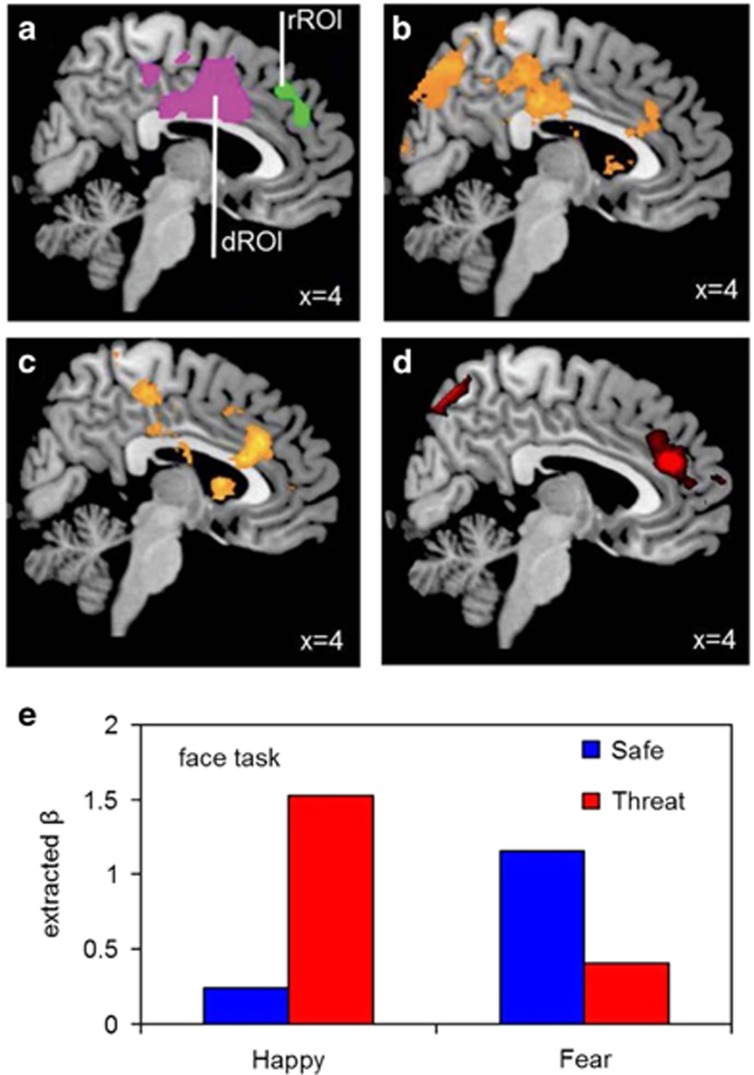
(**a**) Regions-of-interest (ROI adapted from the study by Robinson *et al.*^6^) showing the dorsal (dROI) and rostral (rROI) medial cortical clusters encompassing medial/anterior cingulate and medial (pre)frontal cortical regions. Significant (**b**) dorsal task × shock-threat × valence interaction in amygdala coupling across the whole brain is driven by (**c**) replicated shock-threat × valence (that is, ‘affective bias-related') coupling interaction in face task. By contrast, attending to shapes over emotional faces engages (**d**) ‘attentional control-related' coupling between the amygdala and a rostral portion of the medial prefrontal/cingulate cortex (inference was performed in ROIs, but contrasts are whole-brain contrasts for illustrative purposes). The interaction during the face task was driven by (**e**) increased threat-potentiated coupling during happy, but not fearful faces (betas extracted from the identified dROI posterior medial cortical peak (*xyz*=6,−32,56) for illustration purposes).

All inference was based on voxel-wise region-of-interest (ROI) analyses. Specifically we used more dorsal (dROI) and more rostral (rROI) ROIs generated from our first shock-threat imaging paper^5^ (and used since^6, 7^). These ROIs (Figure 2a; adapted from the study by Robinson *et al.*^6^) are freely available for download (http://dx.doi.org/10.6084/m9.figshare.1040417) and were generated from the peak shock-threat × valence connectivity clusters, which were centred on the medial wall of the cingulate and (pre)frontal cortices. The dROI comprises a large cluster (20 312 mm^3^) for which the original peak was *xyz*=2,2,40. It encompasses anterior and posterior regions of the midcingulate cortex^35^ and (pre) supplemental motor area extending into dorsal medial prefrontal cortex. The smaller rROI (4024 mm^3^) represents a cluster for which the original peak was *xyz*=8,34,50 and comprises largely medial prefrontal cortex, extending into the anterior cingulate cortex.^35^ ROI analyses were completed with family-wise error (FWE) correction for multiple comparisons. In the results, this is listed as ‘p(FWE_peak_SVC)' which stands for ‘family-wise error-corrected voxel-wise *P*-value within the small volume corrected ROI'. Where the p(FWE_peak_SVC) is at trend level for simple effects making up interactions, small volume corrected *P*-values are also listed as ‘p(SVC)'.

## Results

### Manipulation check

Subjects rated themselves significantly more anxious during the threat blocks (mean rating: 5/10) relative to safe blocks (mean rating: 2/10; F(1,35)=69, *P<*0.001, *η*^2^=0.7). Bayesian analysis confirmed that the model with the highest evidence relative to the null out of all possible models comprised a main effect of threat only (logBF10=48.4). Note that complete rating data was lacking for seven subjects. Restricting analysis to the first block limited missing data to three subjects and resulted in the same main effect of condition (F(1,39)=104, *P<*0.001, *η*^2^=0.7)).

### Behavioural performance

As predicted there was a task × shock-threat × valence interaction in reaction time (F(1,42)=5.9, *P*=0.019, *η*^2^=0.12). Bayesian analyses indicated a winning model comprising main effects of task, shock-threat and valence and a task × shock-threat interaction (logBF10=96.1). However, this was no more predictive (that is, dividing the larger by the smaller BF10s resulted in a number <3 (ref. 32)) than the model also including a task × shock-threat × valence interaction (logBF10=95.8). This reaction time interaction was seen in the absence of a comparable interaction in error rates (F(1,42)=1.9, *P*=0.17, *η*^2^=0.044; logBF10=-9.6).

Breaking the three-way interaction down by task, Bayesian analyses confirmed that the winning model for the face task comprised main effects of valence, shock-threat and shock-threat × valence interaction (logBF10=13), whereas the winning model for the shape task comprised the main effects of shock-threat and valence only (logBF10=8.8; Figure 1b). In other words, we replicated a threat × valence interaction in the face task, and showed that it was abolished in the shapes task. Frequentist analyses confirmed a shock-threat × valence interaction in the face task (F(1,42)=19, *P<*0.001, *η*^2^=0.31), but only main effects of shock-threat (faster response under threat: F(1,42)=12, *P*=0.001, *η*^2^=0.27) and valence in the shape task (faster response to shapes overlaid with happy faces F(1,41)=12, *P*=0.001, *η*^2^=0.22; Figure 1c). The shock-threat × valence interaction in the Face task was driven by opposite effects of shock-threat on the happy (F(1,42)=7.8, *P*=0.008, *η*^2^=0.16), and fear (F(1,42)=4.5, *P*=0.040, *η*^2^=0.1) conditions (Figure 1d). In addition to these within-task effects, subjects were slower overall in the faces (697±9 ms) relative to the shapes (642±11 ms) task (F(1,42)=53, *P<*0.001, *η*^2^=0.56) and more accurate in the shape task relative to face task (winning model of main effect of task: F(1,42)=10, *P<*0.003 *η*^2^=0.19; logBF10=5.7)

### Functional imaging

#### Connectivity analysis

Amygdala-dorsal prefrontal ‘affective bias-related' coupling only engaged when emotional stimuli are attended: We found a number of predicted task × shock-threat × valence amygdala connectivity peaks in our *a priori* dROI (*xyz*=6,−30,52, *T*=4.1, p(FWE_peak_SVC)=0.018/*xyz*=−8,14,34, *T*=3.2, p(SVC)=0.001; Figure 2b). Breaking this down by task—consistent with both predictions and behaviour—there was a shock-threat × valence amygdala connectivity peak in the dROI (*xyz*=6,−28,54, *T*=4.69, p(FWE_peak_SVC)=0.002) (Figure 2c). By contrast, similarly consistent with behaviour, there was no shock-threat × valence amygdala connectivity interaction in either dROI or the rROI (or across the whole brain) during the shapes task when the emotional stimuli were distractors (even at liberal statistical thresholds). There was also no main effect of shock-threat or main effect of valence in amygdala-dROI connectivity during the shapes task. Thus, the amygdala-dROI coupling mechanism is only engaged when emotional stimuli are targets.

Breaking down the interaction in the face task revealed a significant increase in amygdala-dROI connectivity under threat relative to safe for happy faces (*xyz*=6,−32,56, *T*=3.69, p(SVC)<0.0001, p(FWE_peak_SVC)=0.079; Figure 2e), but greater coupling for fearful faces under safe relative to threat (*xyz*=4,−10,30, *T*=4.04, p(FWE_peak_SVC)=0.022). Similarly, connectivity was greater for happy relative to fearful faces under threat (*xyz*=10,8,38, *T*=3.75, p(SVC)<0.0001, p(FWE_peak_SVC)=0.059) and subjects showed greater coupling for fearful faces relative to happy faces under safe conditions (*xyz*=−6,16,28, *T*=4.55, p(FWE_peak_SVC)=0.003).

‘Attentional control-related' amygdala-rostral prefrontal coupling during shapes relative to faces task: There was an overall main effect of task in the rROI, with greater amygdala connectivity when attentional control was putatively engaged to ignore the faces during shapes processing (*xyz*=6,42,24, *T*=4.01, p(FWE_peak_SVC)=0.006; Figure 2d). This effect was not seen in the dROI.

#### Activation analysis

Threat and attention are associated with dissociable prefrontal and amygdala activations: In the non-connectivity, event-related activation analysis, no task × shock-threat × valence activations were seen in our ROIs. There was, however, a main effect of shock-threat in our dROI (*xyz*=6,10,46, *T*=5.72, p(FWE_peak_SVC)<0.0001) and rROI (*xyz*=0,28,54, *T*=5.11, p(FWE_peak_SVC)<0.0001). The right amygdala seed used in the PPI analysis was significantly more active in the faces relative to the shapes task (*xyz*=32,−2,−12, *T*=2.9, p(FWE_peak_SVC)=0.004).

## Discussion

This paper provides evidence that attentional control can modulate activity within anxiety-potentiated amygdala–medial frontal coupling. Specifically, instructing healthy individuals to shift their attention away from emotional stimuli results in a shift in behaviour and a corresponding downregulation of circuit coupling, even while the threatening context and emotional stimuli remain. This downregulation in ‘affective bias-related' coupling was accompanied by a corresponding increase in ‘attentional control-related' coupling in a rostral portion of the medial prefrontal cortex. Taken together, these findings may constitute a mechanism by which attentional control can modulate the neural circuitry of anxiety.

We have previously shown that ‘affective bias-related' amygdala-prefrontal coupling is engaged by threat of shock in healthy individuals.^5, 8^ Here, we extend this to show that healthy individuals can successfully prevent engagement of this threat-specific mechanism, even when threat is still present, when they are instructed to disengage from emotional components of stimuli (as evidenced by significant neural and behavioural effects). This is important because we have shown that this mechanism is chronically engaged in individuals with anxiety disorders and that it varies positively as a function of their self-reported symptoms.^6^ Correspondingly, this study raises the possibility that it may also be possible to ultimately downregulate coupling within this circuit (and hence eventually symptomatology) in patient populations through psychological instruction. Critically, this observation is consistent with the attentional hypothesis of anxiety^19, 36^ which posits that healthy individuals are able to use attentional control to downregulate anxiety responses and that individuals with poor attentional control are particularly susceptible to anxiety.^20^ By extension, we would predict that exploring this same paradigm in individuals with anxiety disorders prior to treatment would reveal a corresponding propensity to engage this coupling even when instructed to attend non-emotional shape stimuli in an threatening context.^19, 36^ A question for future research, therefore, is whether it is possible to re-establish control over this circuitry in anxiety disorders. One potential avenue of interest, for instance, might be attempting to enhance the impact of psychological instruction in patients through the use of cognitive enhancers (for example, methylphenidate or modafinil).^37^ This has precedent in the use of d-cycloserine to facilitate extinction training in translational models of anxiety disorders.^38^

Interestingly, as highlighted in the introduction, meta-analyses of attentional control^9^ and threat or anxiety^14, 15^ reveal overlapping neural substrates. As such it has been argued that attentional control mechanisms dampen anxiety processes by defeating them in a competition for neural processing representation. Our findings do in fact provide some support for this proposition. Specifically, a decline in threat-specific ‘affective bias-related' coupling in our dROI during the shapes task was accompanied by a significant increase in task-specific ‘attentional control-related' coupling in our rROI. Importantly, this latter task-specific coupling does not interact with either threat or valence and as such may serve to dampen the influence of these factors over behaviour (and hence abolish the shock-threat × valence interaction in reaction time). In our first study, coupling between the amygdala and both the dorsal and rostral regions was engaged by threat of shock,^5^ and as such these regions might represent spatially discreet nodes along a single common circuit in which ‘attentional control' over-rides ‘affective bias' for control of this circuitry during the shapes task.^14^

However, it should also be noted that it is the more dorsal of these ROIs that has been most consistently implicated across our studies.^6, 7^ As such, an alternative explanation is that these two clusters constitute nodes on adjacent but separable circuits, with a more rostral ‘attentional control-related' mechanism over-riding the more dorsal ‘affective bias-related' circuit when healthy individuals are instructed to ignore the emotional stimuli. Indeed, previous work has implicated activity in a cluster similar to our rostral region in top–down attentional control over emotional distractors.^23^ One key question for future work, therefore, is whether meaningful functional segregation exists between these regions along the medial wall of the frontal and cingulate cortex and, if so, how these mechanisms are causally related.

It should be noted that in addition to these coupling effects, we also showed differences in event-related activation. We replicate the frequently observed threat-specific dorsal cingulate/medial prefrontal activity increase under threat of shock,^14, 15^ but again demonstrate that this effect is consistent across valence and attention conditions. One possibility is that activity in this region corresponds with the overall threat context^39^ or level of ‘worry' about that threat,^15^ but that other processes (for example, the coupling between this region and the amygdala described above) constitute the behavioural instantiation of response to that threat. Again, however, the relationship between activation and coupling is unknown and future work is required to establish direction and causality.

The current results also raise the possibility that the exact nature of the behavioural effect instantiated by amygdala–medial frontal coupling is subject to additional moderators. Specifically, although we replicate threat-specific amygdala–medial frontal coupling, the shock-threat by valence interaction was driven by happy faces in this study (alongside a corresponding behavioural effect on the happy faces). While unanticipated, there may be a relatively simple explanation for this. Specifically, in our first study threat of shock induced negative bias via reduced reaction time to fearful faces,^5^ while in the present study negative bias was instantiated by increased reaction time to happy faces. In both cases, the effect is to tip the scales of affective processing towards fearful, and away from happy faces. Indeed pooling data from prior studies in a mega analysis (see Supplementary Materials) shows that threat can significantly push behaviour in both directions to drive negative bias. Additional, as yet unclear, moderators (such as context, task demand or noise) may determine whether emotion-specific effects are a result of a pull towards fearful faces (that is, more anxiety/fear-like behaviour) or a push away from happy faces (that is, more anhedonia-like behaviour) or both.

Importantly, however, across both studies, the trial type on which negative affective bias was most clearly manifest at the behavioural level was the trial type associated with increased threat of shock-potentiated coupling. Thus, as argued above, the ‘affective-bias-related' coupling may underpin behavioural manifestation of threat effects and so is correspondingly seen on the trials showing the greatest threat of shock-potentiated behavioural bias. Evidence that threat of shock-potentiated coupling is also seen on an adapted resting state paradigm in which no trials are presented^8^ provides a clear indication that coupling effects need not be trial or behaviour specific; the particular emotion-specific behavioural manifestation being mediated by context and/or task demands. Thus, threat of shock may instantiate negative affective bias by influencing both positive and negatively valenced trial types, but across all cases effects are driven by threat of shock-potentiated coupling increases. Future work is of course needed to confirm this hypothesis and explore other potential moderating factors. Of note, it is unclear whether the coupling is critically involved in the generation of the attentional bias itself, or whether both coupling and behavioural bias are under the control of an additional process such as attentional control.

A further point to note regarding behavioural effects is that although the anxiety-potentiated behavioural effect was abolished in the shapes task, we do still see a main effect of valence. This indicates that the emotional content of the shapes may have ‘leaked' though. Similar effects have been demonstrated before^26^ thus one hypothesis is that this main effect of valence seen in the shapes task may reflect intact emotional perception, while attenuated shock-threat × valence interaction reflects intact emotional control. Again, this is a speculation and more work is required. Indeed, the observation that the emotional faces influenced neutral shape perception is reminiscent of the emotional Stroop effect (for example, where emotional words influence neutral colour perception). To this end, however, it should also be noted that our effect—speeded response to identify a shape under threat—differs from that seen on a colour/threat-word Stroop task,^27^ in which threat of shock slows word-colour naming (in high-trait anxious individuals).

In summary, we provide evidence that a mechanism implicated in both induced^5, 8^ and pathological^6^ anxiety can be modulated in healthy individuals through simple attentional task demands. We therefore extend our prior work showing that this circuitry can be modulated pharmacologically^7^ to show that it can also be modulated via psychological task instructions. Future work should explore whether it is also possible to modulate circuit activity psychologically in patient populations—perhaps aided by cognitive enhancers^37^—as a means towards understanding and refining treatment outcomes for this debilitating disorder.
