# Supplementary material for: Anxiety-potentiated amygdala–medial frontal coupling and attentional control
Source: Transl Psychiatry. 2016 Jun 7;6(6):e833–. doi: 10.1038/tp.2016.105 (PMC4931603; doi:10.1038/tp.2016.105)
Supplement: Supplementary Information [file tp2016105x1.doc]

**Supplement**

**Post-hoc mega analysis**

***Methods***

The direction of the behavioural effect differed from our prior study so we also ran a post hoc mega analysis (N=97) of the reaction time to face stimuli in this paper, our original paper(5) and an unpublished dataset collected at University College London (Data available online: https://dx.doi.org/10.6084/m9.figshare.2198995). Here we use mega-analysis to simply denote that all data from all subjects is included in the analysis as opposed to a single collapsed summary statistic from each study (as in meta-analysis).

***Results***

The pooled mega analysis confirmed the shock-threat*valence interaction (F(1,96)=26,p<0.001,η^2^=0.21) but revealed that it was driven by significant and opposite effects of shock-threat on happy and fearful faces. Specifically shock-threat speeded response to fearful faces (F(1,96)=11,p=0.001,η^2^=0.10), but slowed response to happy faces (F(1,96)=6,p=0.01,η^2^=0.06). Bayesian analysis confirmed that the ‘winning’ model (logBF10=25.5) included a shock-threat*valence interaction alongside a valence*study interaction reflecting the effects across studies.
